# Shaping pre-modern digital terrain models: The former topography at Charlemagne’s canal construction site

**DOI:** 10.1371/journal.pone.0200167

**Published:** 2018-07-05

**Authors:** Johannes Schmidt, Lukas Werther, Christoph Zielhofer

**Affiliations:** 1 Institute of Geography, Leipzig University, Leipzig, Germany; 2 Seminar of the Archaeology of Prehistory to the Early Middle Ages, Friedrich-Schiller University, Jena, Germany; University at Buffalo - The State University of New York, UNITED STATES

## Abstract

The use of remote sensing techniques to identify (geo)archaeological features is wide spread. For archaeological prospection and geomorphological mapping, Digital Terrain Models (DTMs) on based LiDAR (Light Detection And Ranging) are mainly used to detect surface and subsurface features. LiDAR is a remote sensing tool that scans the surface with high spatial resolution and allows for the removal of vegetation cover with special data filters. Archaeological publications with LiDAR data in issues have been rising exponentially since the mid-2000s. The methodology of DTM analyses within geoarchaeological contexts is usually based on “bare-earth” LiDAR data, although the terrain is often significantly affected by human activities. However, “bare-earth” LiDAR data analyses are very restricted in the case of historic hydro-engineering such as irrigation systems, mills, or canals because modern roads, railway tracks, buildings, and earth lynchets influence surface water flows and may dissect the terrain. Consequently, a "natural" pre-modern DTM with high depth accuracy is required for palaeohydrological analyses. In this study, we present a GIS-based modelling approach to generate a pre-modern and topographically purged DTM. The case study focuses on the landscape around the Early Medieval *Fossa Carolina*, a canal constructed by Charlemagne and one of the major medieval engineering projects in Europe. Our aim is to reconstruct the pre-modern relief around the *Fossa Carolina* for a better understanding and interpretation of the alignment of the Carolingian canal. Our input data are LiDAR-derived DTMs and a comprehensive vector layer of anthropogenic structures that affect the modern relief. We interpolated the residual points with a spline algorithm and smoothed the result with a low pass filter. The purged DTM reflects the pre-modern shape of the landscape. To validate and ground-truth the model, we used the levels of recovered pre-modern soils and surfaces that have been buried by floodplain deposits, colluvial layers, or dam material of the Carolingian canal. We compared pre-modern soil and surface levels with the modelled pre-modern terrain levels and calculated the overall error. The modelled pre-modern surface fits with the levels of the buried soils and surfaces. Furthermore, the pre-modern DTM allows us to model the most favourable course of the canal with minimal earth volume to dig out. This modelled pathway corresponds significantly with the alignment of the Carolingian canal. Our method offers various new opportunities for geoarchaeological terrain analysis, for which an undisturbed high-precision pre-modern surface is crucial.

## Introduction

It is obvious that the present relief does not match the pre-modern topography. The present relief is often detected on a large scale via LiDAR (Light Detection and Ranging), whereas the pre-modern relief needs to be reconstructed. LiDAR is used to scan the surface from the air by measuring the optical distances and velocities of laser beams. Airborne Laser-Scanning (ALS) LiDAR provides direct measurements of vegetation cover (first pulse) and “bare earth” (last pulse), resulting in a 3D point cloud. Due to the penetration of the light signal through vegetation cover, it is possible to detect the topography and archaeological features under light forest canopy [1,2]. By filtering the data, vegetation cover can be removed so that the ground surface is displayed in the terrain model [1,3].

According to Web of Science [4], the number of publications referring to LiDAR data in archaeological and environmental science has been growing exponentially since the mid-2000s, and the availability and successful application of these datasets have increased. From a geoarchaeological point of view, LiDAR offers a fast, non-destructive tool for remote sensing and large-scale prospection that provides valuable information about the location and extent of anthropogenic surface structures [5–7,2,8]. Nevertheless, the method can only document the present relief, which is usually profoundly modified by human activity and does not match with the pre-modern relief. Furthermore, the relief has been modified significantly by extensive linear and non-linear structures such as settlements and infrastructure (roads, railway lines, ditches, etc.), especially in the modern era. All of these structures alter the natural landscape surface, especially with regard to palaeohydrological interpretability.

In recent years, there have been several approaches to reconstruct pre-modern terrain based on the interpolation of large datasets from drillings, archaeological excavations, and outcrops [9,10]. Other studies combine archaeological excavation data and geophysical data to interpolate detected pre-modern surface heights [11–13]. Both approaches are inductive methods based on field data interpolation and elaborate (geo-)archaeological and geophysical fieldwork, and post-processing. There are also deductive approaches to model and reconstruct the palaeo-terrain via the digital deconstruction of present LiDAR-derived Digital Terrain Models (DTMs) [14–17].

**Fig 1:**
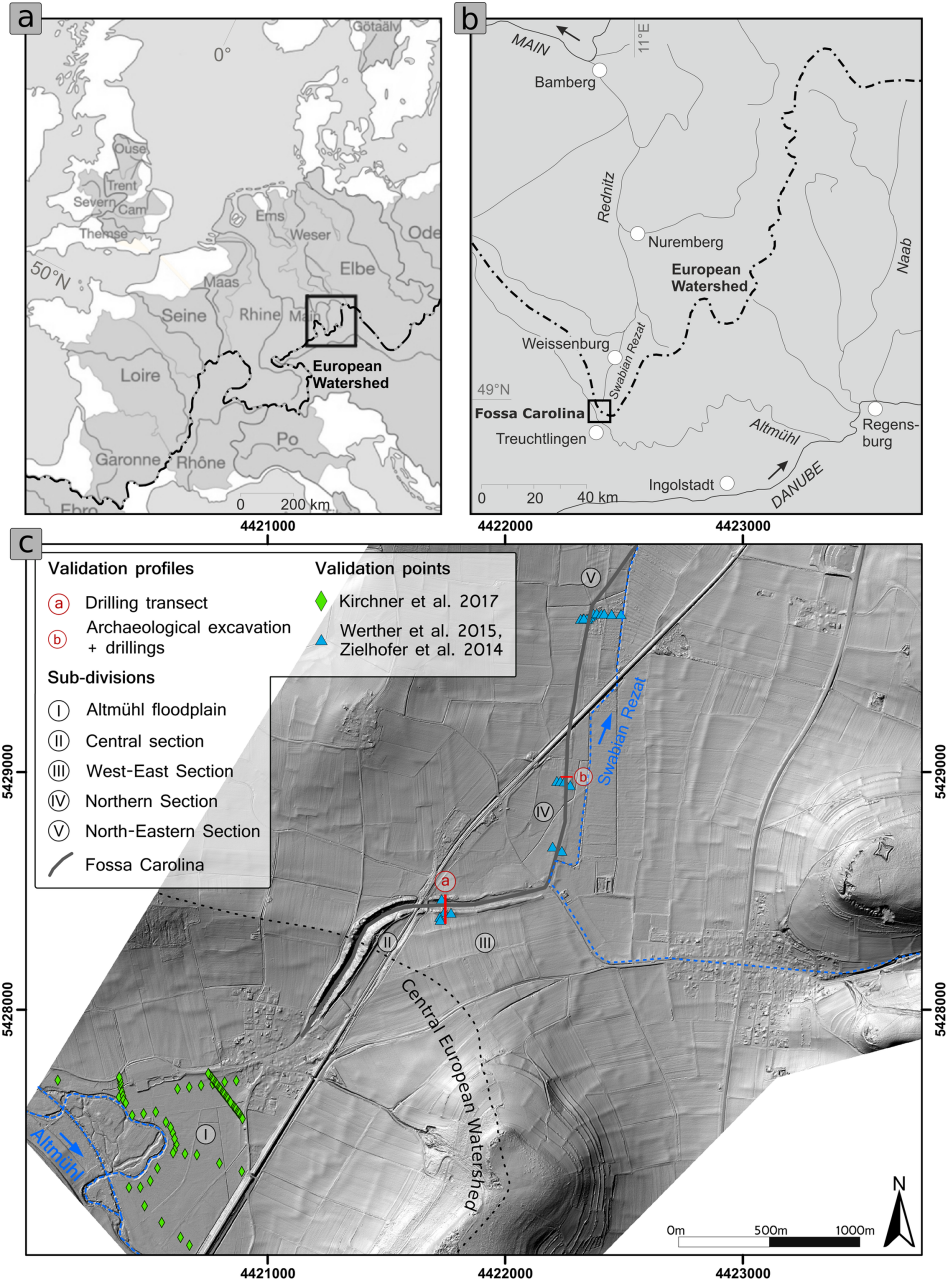
Geographical setting of the study area on different scales. a) Central European setting in relation to main drainage basins and the Central European Watershed. b) Regional setting of the Fossa Carolina in relation to tributaries of the Rhine-Main drainage system and the Danube drainage system. c) Local setting of the Fossa Carolina (hillshade) with five sub-divisions [28] and ground-truth validation points. LiDAR data have been provided by the Bavarian State Office for Land Surveying.

Our case study examines Charlemagne’s summit canal, or *Fossa Carolina*, an Early Medieval hydro-engineering project to bridge the Central European watershed (see Fig 1 and S1 Fig). The canal was built on the order of Charlemagne in 793 AD to connect the drainage basins of the Rhine-Main system and the Danube system to create a navigable waterway [18–20].

In the Carolingian period, inland navigation was very important for mobility, communication, military operations and economic exchange in the expanding Frankish empire [19,21–24]. After a long period without any large canal construction schemes from 3^rd^ to 8^th^ century AD, the *Fossa Carolina* is the first post-Roman large-scale canal North of the Alps [19,25,26,24].

It is the only pre-modern attempt to bridge the Central European watershed, which was at least partially finished – a Roman attempt in the 1^st^ century AD did not go beyond the planning phase [19]. Bridging this watershed was crucial, because it was a serious obstacle for mobility between different drainage systems [19,20,27]. To transport cargo, passengers and sometimes also ships from one river catchment to another, terrestrial routes had to be used if there was no canal. These portages have been bottlenecks, because they caused an enormous additional effort due to transhipment [20,25,27].

Furthermore, the *Fossa Carolina* is also one of the most significant artificial terrain modifications of the Early Middle Ages [24]. It was planned as a summit canal due to the different levels of both tributaries [28,19]. Nevertheless, the hydrological concept and the reasons for the implemented pathway remain unclear due to the lack of a high-precision model of the topography from the time of construction.

We present a GIS-based modelling approach to improve and purge a present DTM on a sub-landscape scale in the surroundings of the *Fossa Carolina*. The DTM is based on LiDAR data with a spatial resolution of 1x1 m. We revised the model by eliminating all kind of detectable disturbing factors such as roads, railway lines, buildings, Carolingian features, present and historic cadastre boundaries, and other human features such as sewage plants, rain retention basins, etc. Furthermore, we use levels from geoarchaeological drillings and archaeological excavations to ground-truth the model and to validate the reconstructed pre-modern surface.

## Study area

The 12.5 km^2^ study area is located in the range of the Southern Franconian Jura foothills in Bavaria, Southwest Germany (Fig 1). The bedrock of the escarpment consists of bedded Upper Jurassic limestone, whereas the parent material of the foothills consists of Middle Jurassic claystone and Upper Pleistocene sandy valley fills [29]. The study area is part of the Central European watershed and locally features two sub-drainage systems (Fig 1b).

The Altmühl River is a tributary of the Danube and drains towards the Black Sea. In the study area, the Altmühl floodplain with wide meander loops and a modern straightened watercourse are typical elements of the landscape [30]. In contrast, the Swabian Rezat River (Fig 1b) is part of the Rhine-Main catchment and drains towards the North Sea. The Rezat fen is located along the upper course of the Swabian Rezat River and consists of thick organic sediments. In modern times, the Swabian Rezat has been straightened and moved from the natural riverbed, and it is difficult to precisely detect the natural riverbed via DTM or aerial images. The European watershed divides both sub-drainage systems and trends as a shallow valley ridge that mainly consists of sandy to loamy fluvial deposits from the Late Pleistocene age.

**Fig 2:**
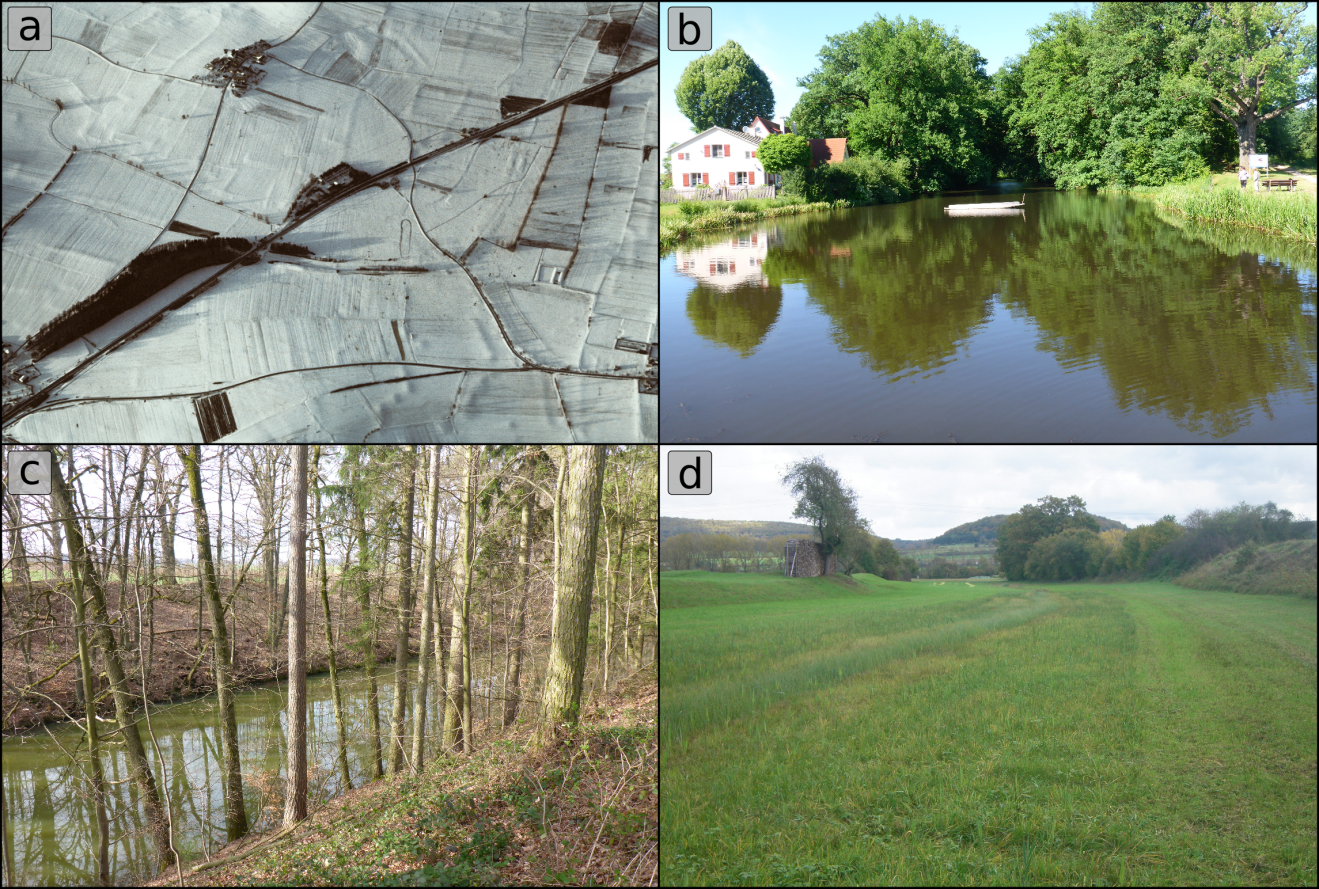
Photographs of Charlemagne’s summit canal. a) Aerial image of the canal with shadow marks highlighting terrain differences [31], b) Pond at the southern edge of the canal in Graben (Photo: Werther 2017), c) Central section with afforested ramparts (Photo: Werther 2017), d) West-East-section (Photo: Leitholdt 2014).

The course of Charlemagne’s summit canal could be divided into five different sections, according to their geographical and geoarchaeological conditions [28] (Figs 1c and 2). The southernmost canal remains are located in the village Graben and are still visible as a pond (Fig 2b). The central section is characterized by afforested ramparts (up to 13m above present pond level; Fig 2c). In the West-East-section, the ramparts are lower and the former canal is silted (Fig 2d). The Northern and North-Eastern section are marked by relatively flat ramparts and only hardly visible at the surface.

## Material and methods

### Basic data

The basic digital data for our approach are a LiDAR DTM (1x1 m spatial resolution, height accuracy of ±0.2 m); a contemporary high-resolution orthophoto (0.2-m spatial resolution); a contemporary high-resolution land use vector layer with buildings, roads, railway lines, and present cadastre boundaries (Bavarian State Office for Land Surveying and Open Street Map); and the first high-resolution historical cadastre map from 1820–1822 AD, which includes cadastre boundaries and roads from before modern land consolidation (provided by the Bavarian State Conservation Office, Table 1).

**Table 1:** Basic data acquisition and quality.

	Present cadastre and land use layers	Historical cadastre sheet	Land use layer Open Street Map	LiDAR DTM	Digital Orthophoto (DOP 20)
**Metadata**	[32,33]	[34]	[35]	[36]	[37]
**Source/version**	2012-11-06, provided by LDBV Bayern	2012-11-206 provided by LDBV Bayern; Original maps 1820–1822	2016-05-31, Download	2012-11-06, 2013-08-08, provided by LDBV Bayern	2012-11-06, provided by LDBV Bayern
**Resolution/scale**	1:1000	1:5000	not homogenous	1m	0.2m
**Format**	shapefile	georeferenced tif	shapefile	ascii file, transferred in a DTM raster file	georeferenced tif
**Accuracy**	official - high	official - medium	open data -medium (accuracy verfied based on high-accuracy LiDAR DTM and Digital Orthophotos)	official - high	official - high
**Percentage of the comprehensive layer of anthropogenic structures**	c. 75%	c. 20%	c. 5%		

### Modelling approach

We separated the land use vector layer from the Bavarian State Office for Land Surveying into different thematic shape layers (buildings, roads, railway lines, and present cadastre boundaries). Different types of anthropogenic structures reveal specific spatial impacts on the topography. For example, railway lines have a broad spatial impact along their course because they have wide ballast beds, which disrupt the present topography.

**Fig 3:**
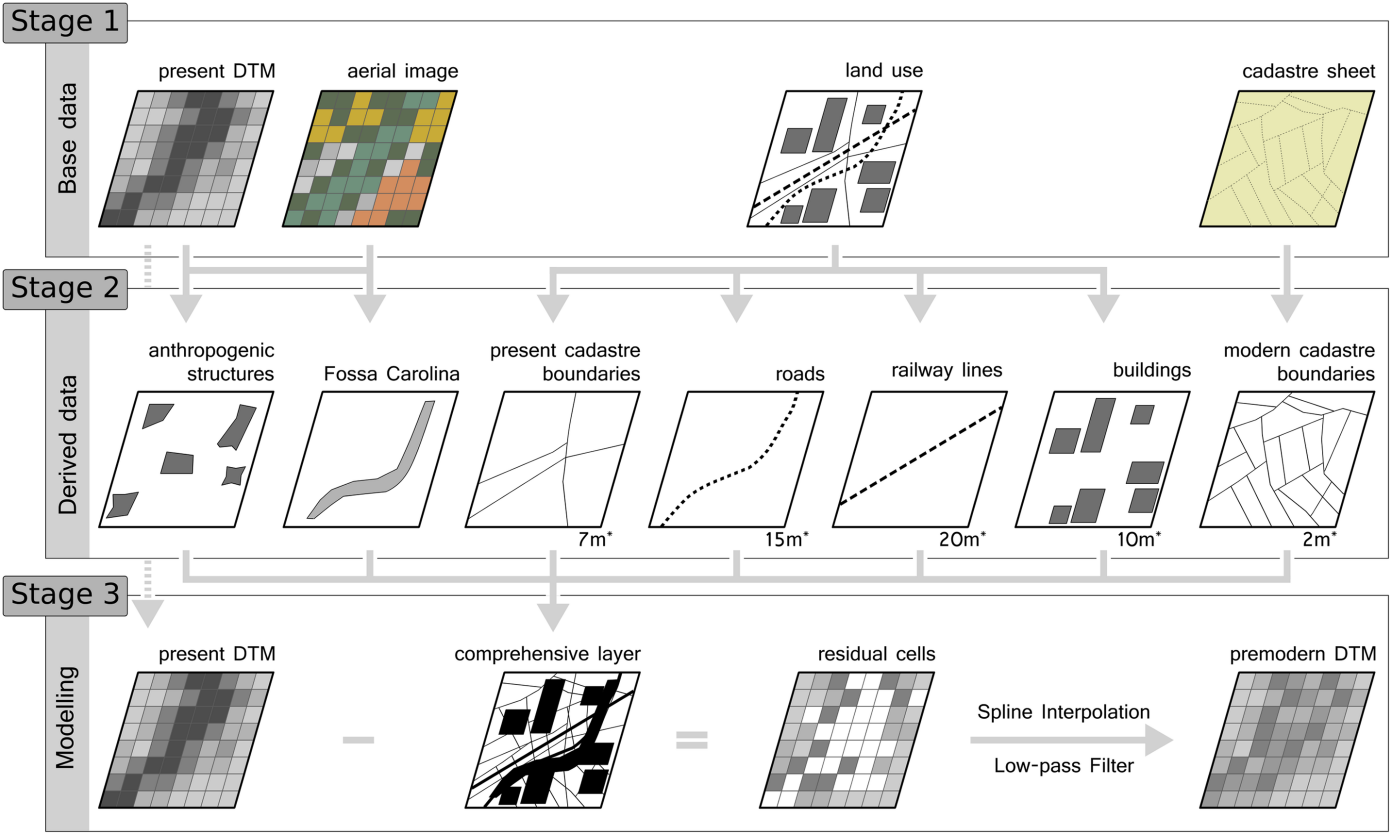
Flowchart of modelling approach in three steps. 1.) Base data acquisition (recent LiDAR DTM, aerial images, shapes of recent land use, historical map), 2.) Deriving specific data layers (* compound specific buffer widths), 3.) Creating a comprehensive buffer layer, removing the affected cells from the modern DTM, interpolating and filtering the residuals.

Aerial images, the land use vector layer from Open Street Map, and LiDAR-based DTM were used to map additional structures such as archaeological features (e.g. *Fossa Carolina*) and industrial features (e.g. sewage plants and quarries) as polygons. Furthermore, we manually digitized the historical cadastre boundaries from the first edition of cadastre sheets at a scale of 1:5,000. Hence, we created structure-specific buffers based on empirical knowledge (manual measurement of topographic impacts of each structure type; Table 2, Fig 3). Subsequently, we merged all buffer layers into one comprehensive layer (S2 Fig). This comprehensive layer is used as a template to remove all detected cells with an anthropogenic impact from the LiDAR-based DTM. This procedure creates a perforated data layer. We interpolated the resulting residual points via a multilevel B-spline. This polynomial function allows for the creation of a continuous and consistent topography and is suitable for unregularly spaced points [38].

**Table 2:** Compilation of compound-specific buffer widths.

	Railway lines	Roads	Buildings	Present cadastre boundaries	19^th^ century cadastre boundaries
Buffer widths	20 m	15 m	10 m	7 m	2 m

**Fig 4:**
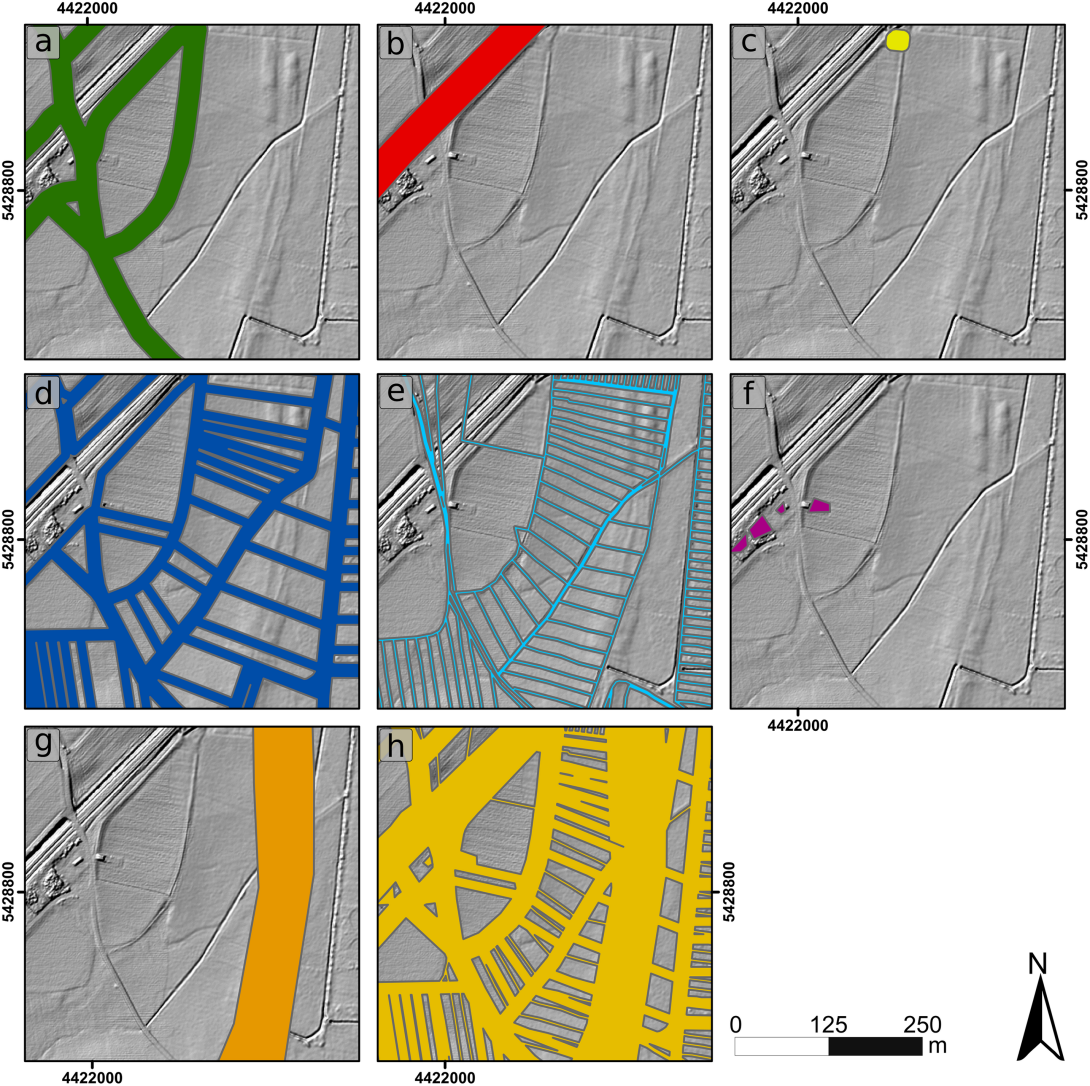
Interim results of the derived data from stage 2 of the modelling approach (see Fig 3). a) Roads, b) railway lines, c) buildings, d) cadastre boundaries derived from present land use layer, e) cadastre boundaries derived from the 1st edition of cadastre sheets, f) additional anthropogenic structures derived from LiDAR-based DTM and aerial image, g) Fossa Carolina, h) comprehensive layer.

Finally, we smoothed the model with a low-pass filter. This procedure adjusts small residuals from the interpolation. The purged DTM with a spatial resolution of 1x1 m no longer contains any larger anthropogenic surface structures and represents the pre-modern topography. Fig 3 shows the stepwise procedure of the modelling approach. The data layer derived from the cadastre sheets is illustrated in the results section (see Fig 4).

### Local-Relief Model

We compared the present and the modelled pre-modern DTM via subtraction of both layers. This tool is known as a Local-Relief Model (LRM) [14]. The resulting raster layer in a two-colour stretch visualises if there is a positive or a negative change in elevation between both datasets. This tool can be used to quantify the erosion, removal, accumulation, and aggradation.

### Validation of the modelled pre-modern DTM

Validation of the modelled pre-modern DTM is crucial for interpretation of the results. Our quantitative approach is based on the comparison of the modelled pre-modern DTM with measured levels of buried soils and surfaces that have been recovered by geoarchaeological drilling campaigns [28,30] and archaeological excavations [39] (Fig 1c). The deviations are computed as the Root Mean Square Error (RMSE) and show the overall error.

#### Least cost path analysis

The Least Cost Path Analysis (LCPA) is a common method for predicting best pathways (with the least cost) between different points. LCPAs are used in archaeology for reconstructing favoured pathways through present and past landscapes [40,41]. At the early stage of the analysis, a criterion has to be selected for the calculation. We have chosen the altitude as a single criterion because we want to predict the most favourable course of the Carolingian canal in consideration of the minimum volume of excavation material required.

We fixed the assumed connection of the *Fossa Carolina* with the present Altmühl and Swabian Rezat Rivers as source and destination points. In this context, we selected the closest position of the Altmühl River to the *Fossa Carolina* because there is no geoarchaeological proof for a link between the Altmühl River and the Carolingian canal [30]. The LCPA results in a line shape, which angular shaped due to raster cells. Thus, we smoothed the resulting line shape for illustrative purposes via iterative averaging [42, 43].

### Hydrogeographical analysis

It is likely that the engineering concept and the course of the *Fossa Carolina* depend on the topographic and hydrogeographic features of the landscape. Therefore, we used hydrogeographic indices for a reliable interpretation of the course of the canal. The Topographic Wetness Index (TWI) indicates potentially wet or dry areas. The calculation is based on the slopes and upstream catchment area. This tool was implemented in SAGA GIS, which provides further descriptions for handling [44].

## Results

### Interim results from stage 2 of the modelling approach

The interim results represent the extracted data from stage 2 (Fig 3) of the modelling approach. The sample extract (Fig 4) shows all layers with their compound-specific buffers. It is obvious that using the comprehensive layer as a mask (Fig 4h) eliminates all larger anthropogenic structures.

### Results from stage 3 of the modelling approach

**Fig 5:**
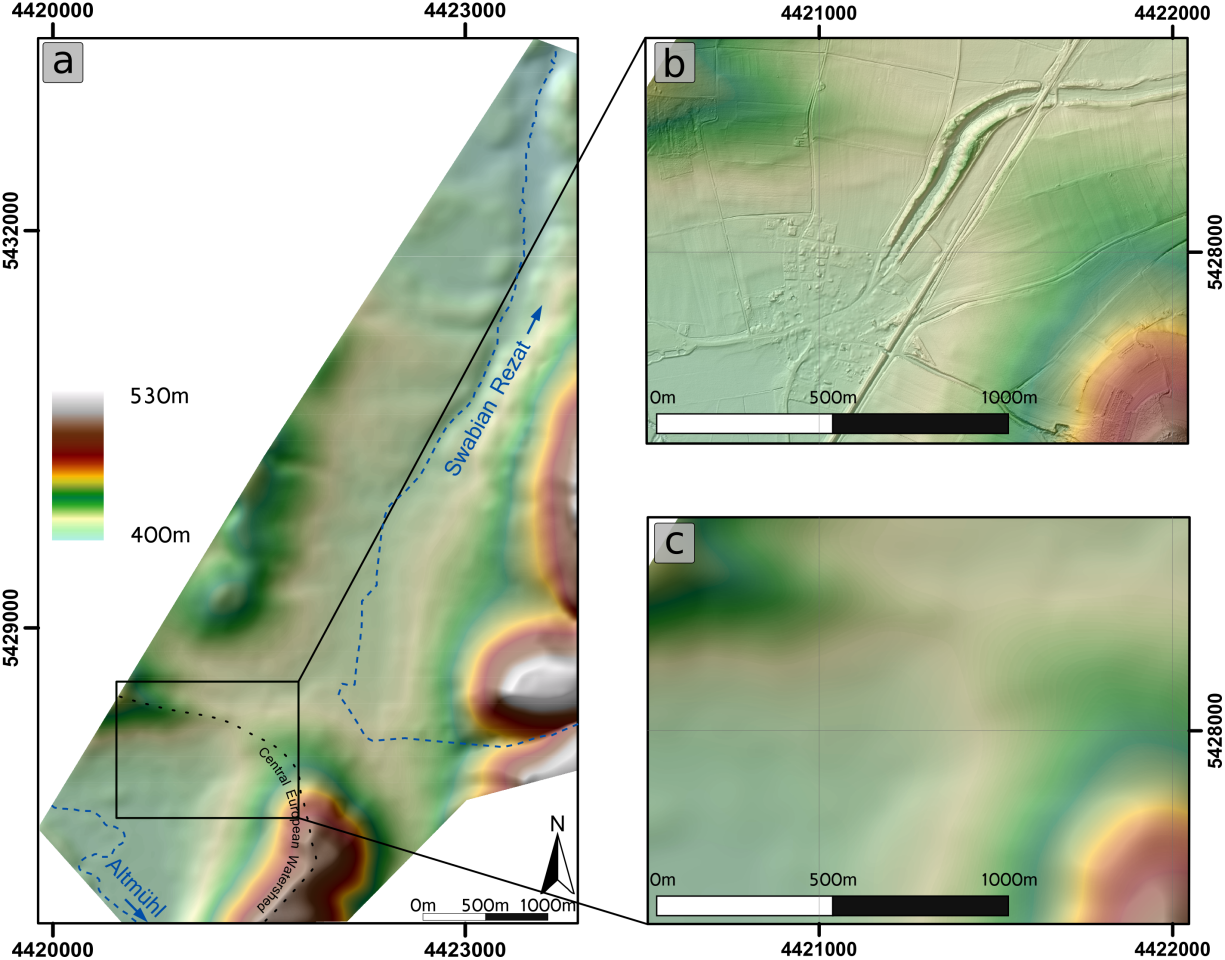
Pre-modern DTM. a) Pre-modern DTM illustrated with hillshade, b) enlarged section with present DTM and hillshade, c) enlarged section with the pre-modern DTM and hillshade.

The modelling procedure results in the pre-modern DTM (Fig 3, Stage 3). The modelled DTM no longer includes larger distinct anthropogenic structures in the study area (Fig 5a and 5c and S4 Fig). The zoomed area in Fig 5b and 5c offers a before-and-after comparison. While the terrain in Fig 5b is dominated by many different anthropogenic structures like buildings, roads, and archaeological features, the terrain in Fig 5c is smooth.

### Model comparison

**Fig 6:**
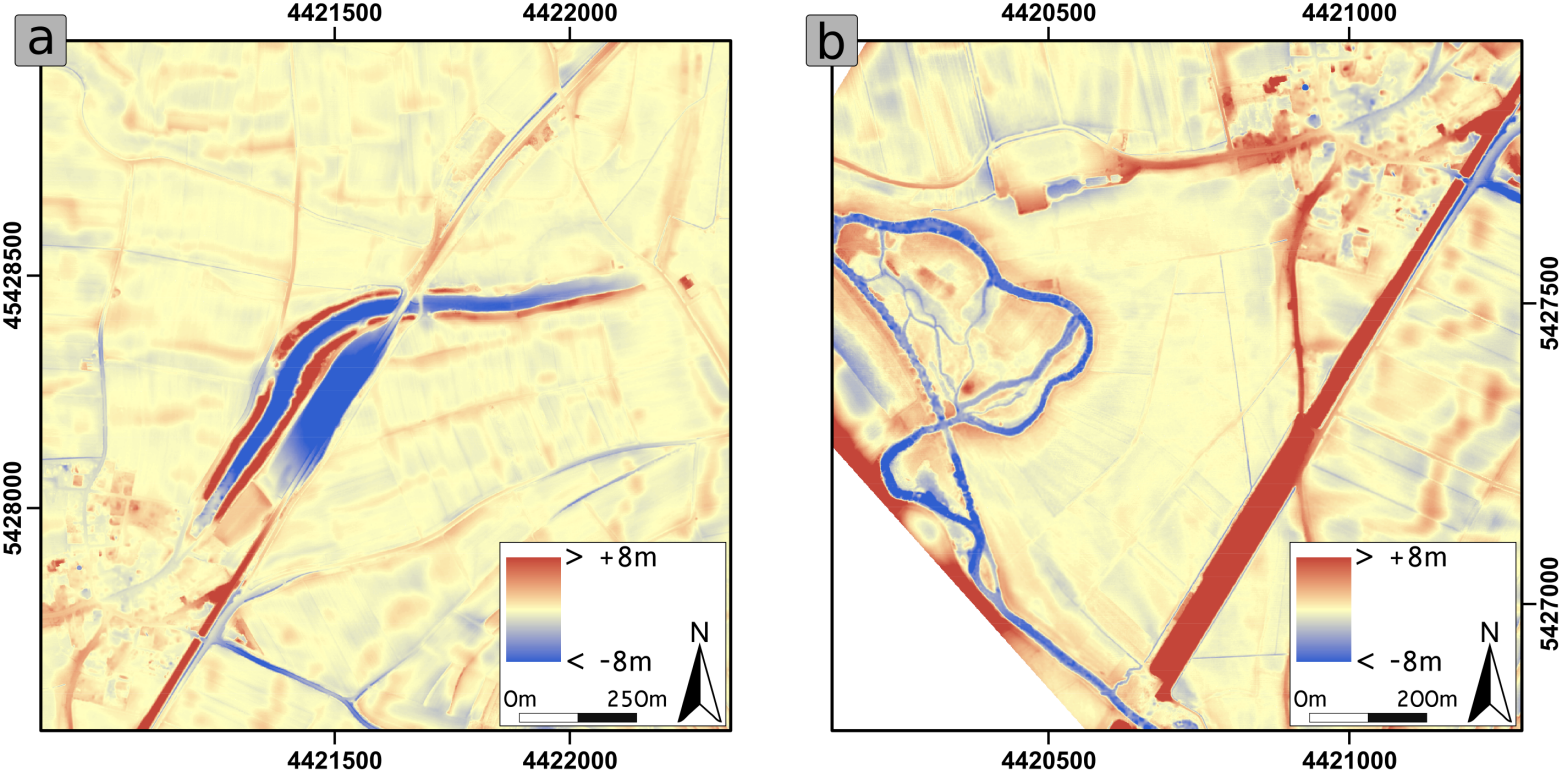
Local Relief Model of the Fossa Carolina central section (a) and the Altmühl floodplain (b). Red indicates sediment aggradation in comparison with the modelled pre-modern DTM; blue indicates sediment removal in comparison with the modelled pre-modern DTM.

The subtraction of the present DTM from the modelled pre-modern DTM results in the LRMs (S3 Fig, Fig 6a and 6b). Both LRMs feature the same colour scale, with red representing aggradation and blue representing the removal of sediment. The LRMs do not differentiate between direct human impact (e.g. buildings, roads) and indirect human impact (e.g. soil-erosion). Fig 6a clearly shows many anthropogenic structures like roads and buildings, the noticeable *Fossa Carolina* dams, and the high-impact railway line. Additionally, many fuzzy structures represent cadastre boundaries or land use boundaries. In Fig 6b, building activities in the area of Graben village and the thick red railway line dominate the eastern and northern parts of the map. The Altmühl floodplain seems to be less affected by human impact, and it is obvious that there are no visible Carolingian canal residuals. Remarkably, distinct levees from the modern age are clearly visible along the Altmühl River.

#### Validation of the modelled pre-modern DTM

Every model has its uncertainties. To determine them precisely, we compared the levels of the modelled pre-modern DTM with measured levels of buried soil surfaces that have been recovered during former drilling campaigns or archaeological excavations. Our validation approach is semi-quantitative because an equal distribution of validation points would be required for a quantitative validation.

**Fig 7:**
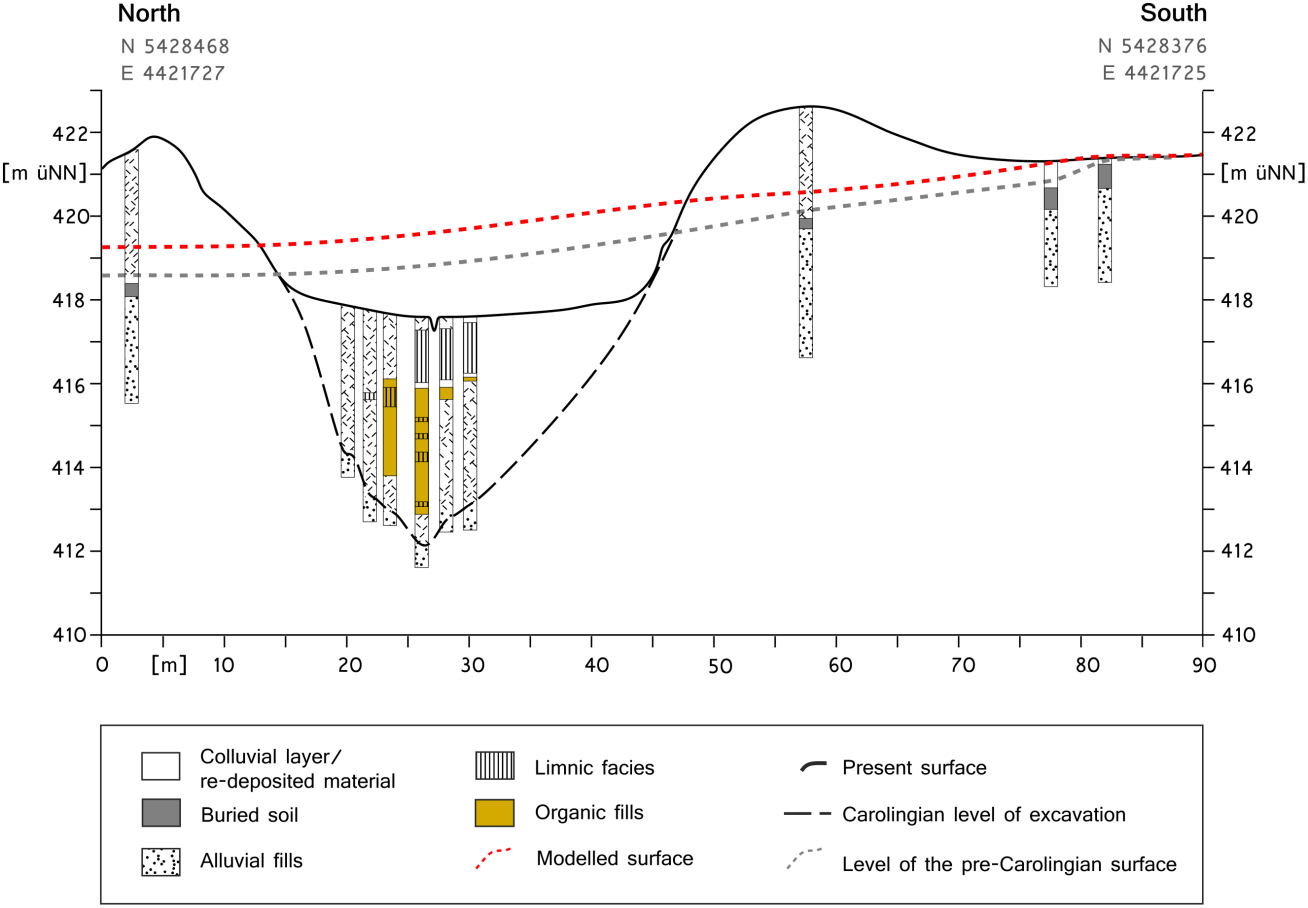
Validation at drilling transect in west-east section of the Fossa Carolina. Drilling data from Zielhofer et al. [28]; the modelled surface is derived from the pre-modern DTM.

The first drilling transect [28] is located in the west-east section of the *Fossa Carolina* (Fig 1c). The drillings gave proof of buried soils under the Carolingian excavation material (Fig 7). The grey dashed line represents the interpolated level of the buried A-horizons, and the red dashed line indicates the surface of the modelled pre-modern DTM. The two lines run parallel but with a slight vertical offset between 35 and 90 cm.

**Fig 8:**
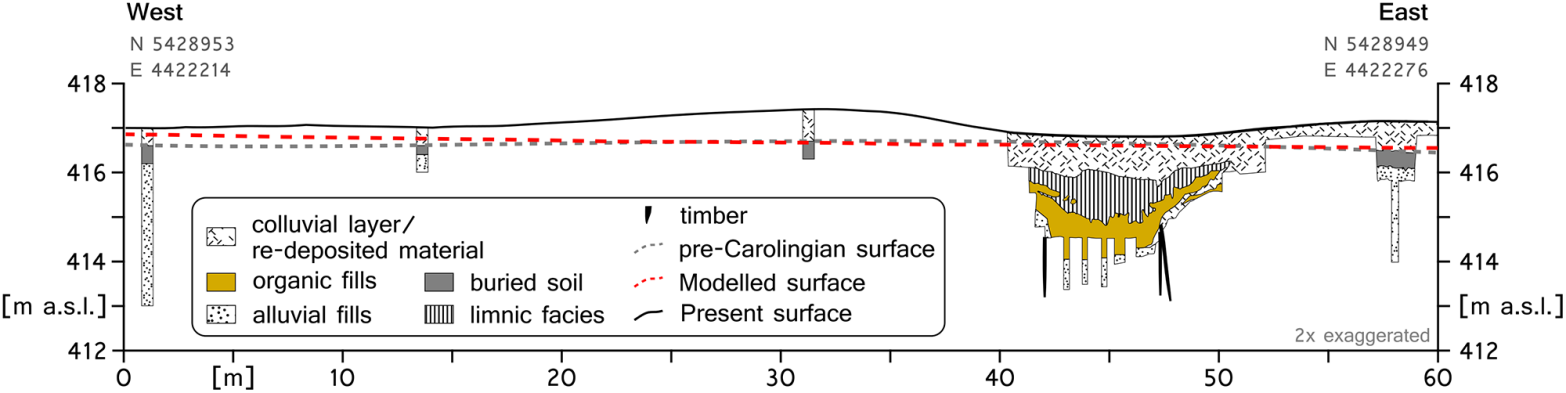
Validation at archaeological excavation site [39] supplemented with drilling data [28] in northern section of Fossa Carolina. The modelled surface is derived from the pre-modern DTM.

The second transect corresponds with an archaeological excavation in the northern section of the *Fossa Carolina* (Fig 1c). The stratigraphy of the archaeological excavation [39,45] and parallel drillings [28] reveal a buried A-horizon in the Rezat fen floodplain (Fig 8). Carolingian excavation material and younger flood loam deposits of around 50 cm cover the pre-modern A-horizon. Here, the surface of the modelled pre-modern DTM has almost no deviation from the buried pre-modern surface.

**Fig 9:**
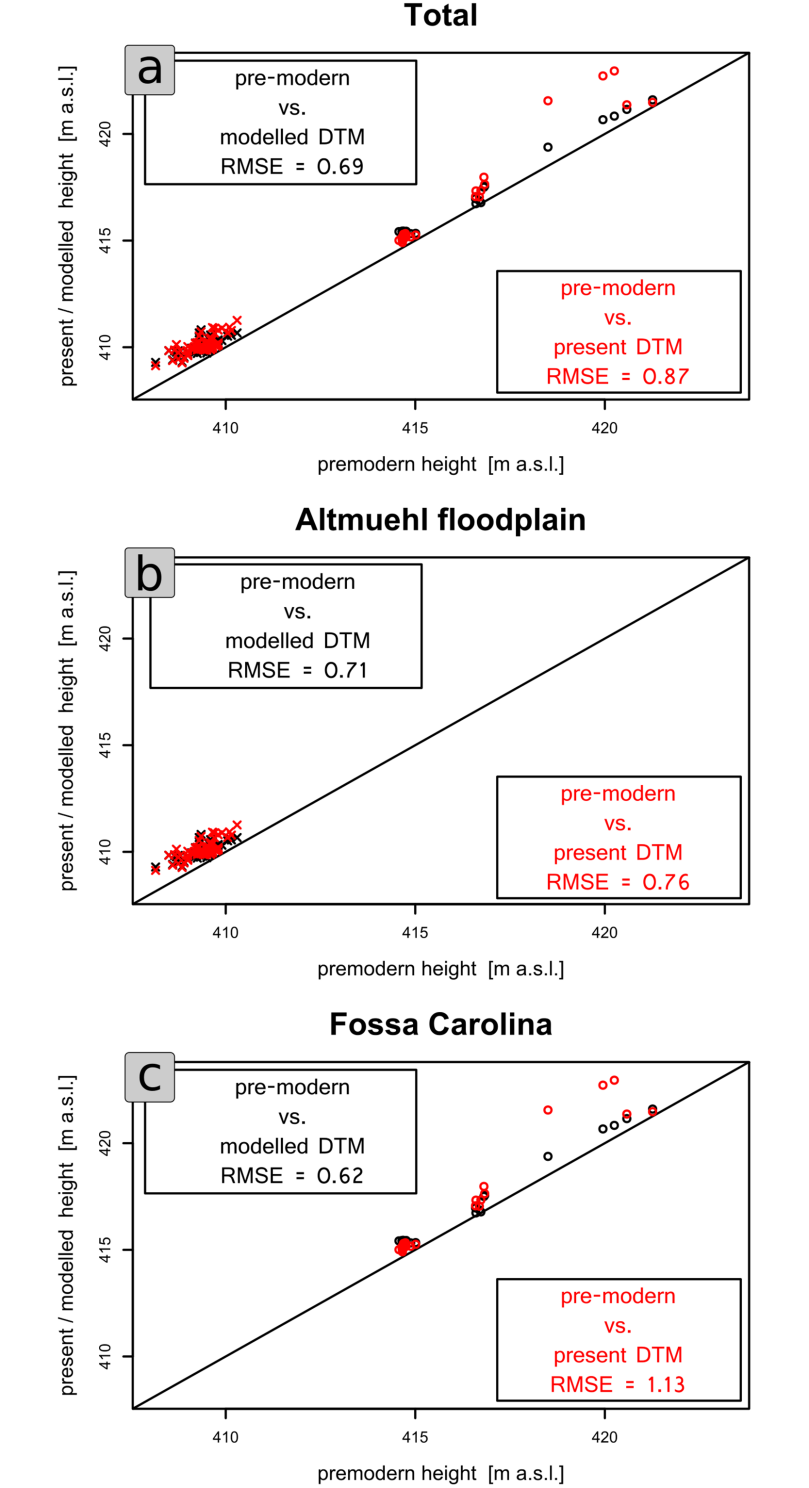
Validation plots of the modelled surface (black dots) and the present surface (red dots) against the observed surfaces (pre-modern height) derived from drillings and excavations. The RMSE (root-mean-square error) is shown in each legend box to estimate the error. a) Total set of all validation points; b) validation points [30] in the Altmuehl floodplain reflecting areas with less anthropogenic overprint (mainly quasi-natural accumulation of alluvial deposits); c) validation from previous studies [39] in the direct surroundings of Fossa Carolina reflecting areas with strong anthropogenic overprint (linear structures).

In order to obtain a quantitative estimation of the error of the modelled pre-modern DTM, we calculated the RMSE (Table 3 and Fig 9). This error is given in metres [46]and represents the mean deviations between the modelled pre-modern DTM and manifold measured levels of pre-modern buried surfaces derived from drillings [28,30] and archaeological excavations [39]. We manually clustered the spatial data in two subsets to estimate the error of different landscape types and associated canal sections. The first cluster includes all data in the direct surroundings of *Fossa Carolina*, reflecting a zone with an intense anthropogenic impact due to the Carolingian canal and railway lines. The second cluster in the Altmühl floodplain reflects an area with generally lower anthropogenic impact on the terrain.

**Table 3:** Comparison of Root Mean Square Errors (RMSE) between the Fossa Carolina and Altmühl validation point clusters and between the measured vs. modelled pre-modern surface and the measured pre-modern surface vs. the present DTM.

	Total	Fossa Carolina	Altmühl floodplain
**Measured (buried) pre-modern surface vs. modelled pre-modern DTM**	0.69	0.62	0.71
**Measured (buried) pre-modern surface vs. present LiDAR-based DTM**	0.87	1.13	0.76
**Improvement [m]**	0.18	0.51	0.05

We calculated the RMSE between the modelled pre-modern DTM and the measured pre-modern surface, as well as between the present DTM and the measured pre-modern surface to estimate the improvement of the modelled pre-modern DTM against the present DTM (Table 3). Generally, there is an improvement of the modelled pre-modern DTM in all areas (0.18 m, Table 3). However, there are noticeable differences between the subsets. The *Fossa Carolina* subset shows a mean improvement of 0.51 m, and the Altmühl subset reveals a mean improvement of only 0.05 m (Table 3). Consequently, there are higher improvements in zones of stronger and more direct human impact on the former topography.

### The modelled pathway of Fossa Carolina

**Fig 10:**
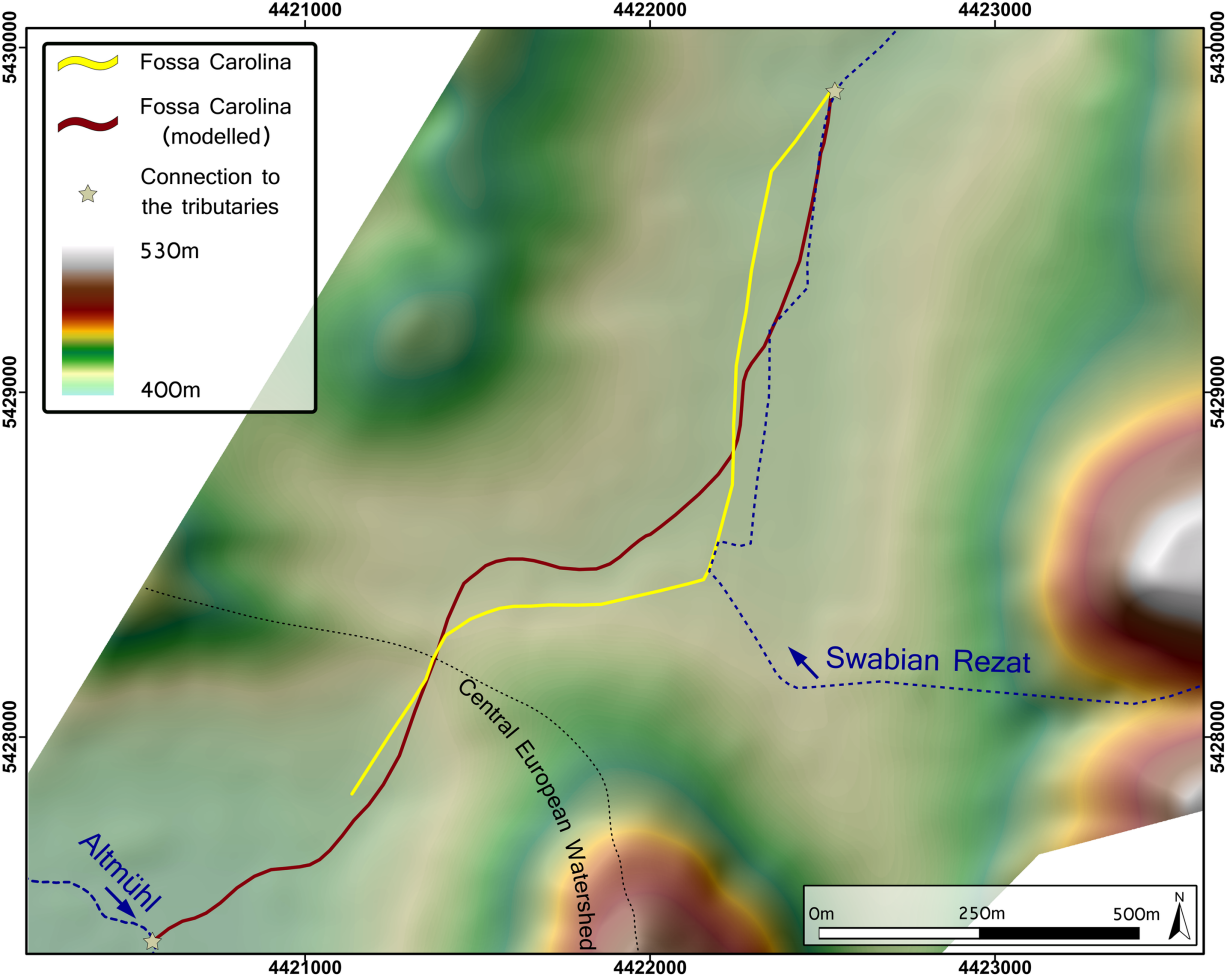
Pre-modern DTM with the Carolingian course of the canal (yellow line) and the modelled course (least cost path analysis).

The LCPA computes the most cost-efficient canal pathway and therefore the minimum earthmoving (Fig 10). In general, it is striking that the modelled canal course follows the real Carolingian canal course quite well (Fig 10). Both courses are S-shaped and indicate an almost identical point for crossing the Central European Watershed. The modelled course has some minor deviations from the real canal course. The first deviation is visible in the west-east section, where the modelled pathway is located more towards the centre of the depression line in the North. The second deviation is detectable in the northern section, where the Carolingian canal course is located slightly more in the west of the modelled pathway.

### Hydrogeographic indices

**Fig 11:**
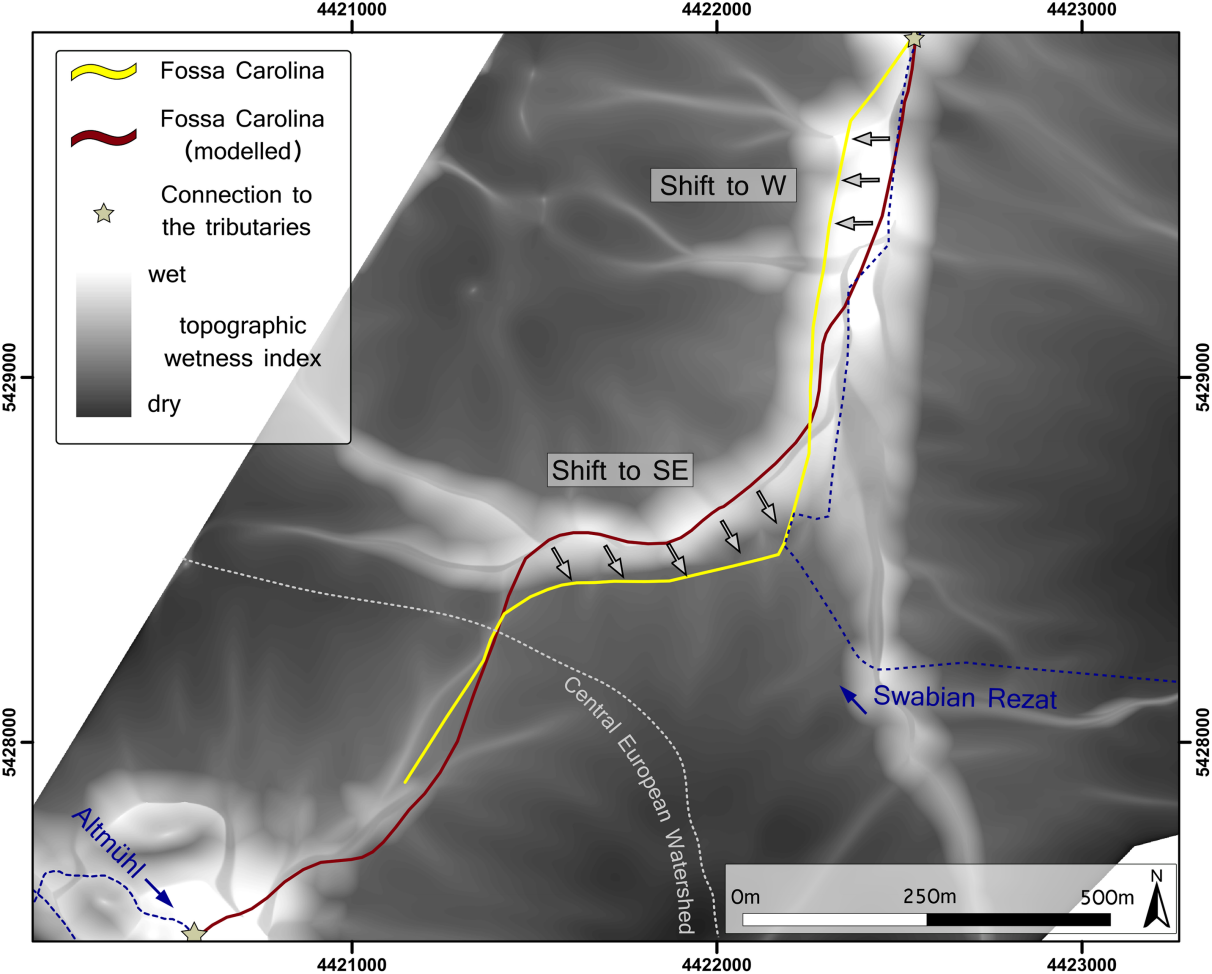
Topographic wetness index with the present course of Fossa Carolina (yellow line) and the modelled course (brown line) based on the pre-modern DTM. Topographic Wetness Index (TWI) in greyscale shows potential wet areas (white colours). Grey arrows show slight deviations between both courses. Blue dotted lines show the present waterways.

The modelled pre-modern DTM allows the calculation of different hydrogeographic indices such as the Topographic Wetness Index (TWI), as disturbing anthropogenic structures that alter the surface runoff have been removed. The modelled wet areas are located in the Altmühl floodplain and in the area of the Rezat fen (Fig 11, S5 Fig). The real course of the *Fossa Carolina* runs at the southern and western margins of the wet areas, whereas the modelled canal pathway runs directly through the wet depression line.

## Discussion

### Palaeo-surface modelling approaches

The modelling of the palaeo-surface is an important tool for archaeological evaluation and geoarchaeological site interpretation [47]. A palaeo-surface can be reconstructed by deductive, inductive, or combined approaches. To evaluate our study in terms of effort, accuracy, applicability, and validation, we compared it with available studies with a terrain reconstruction approach that result in a palaeo-DTM (see Table 4).

**Table 4:** Comparison of modelling approaches in palaeo-terrain research with geoarchaeological issues.

Study area	Dating	Approach	Applicability	Low effort	Accuracy	Validation	Data	Spatial scale	Reference
general study	-	deductive	++	++	-	no	LiDAR DTM	none	*Hesse et al*. *2010*
Karlburg, Germany	-	deductive	++	++	-	no	LiDAR DTM	c. 1 km^2^	*Höfler et al*. *2015*
Sandy Flanders, Belgium	-	deductive	+	+	+	yes	LiDAR DTM, topographical vector data, historic map (1863, 1909)	1400 km^2^	*Werbrouk et al*. *2011*
Gooi and Vechtsdreek area, Netherlands	5 time slices (0 AD, 800 AD, 1,350 AD, 1,885 AD, 2,000 AD)	deductive	+	+	-	no	present DTM	200 km^2^	*van Loon et al*. *2014*
**Fossa Carolina, Germany**	**pre-modern**	**deductive**	**++**	**+**	**+**	**yes**	**LiDAR-DTM, present land use data, historic map, aerial image**	**12.5 Km**^**2**^	***This study***
Scheldt Polders, Belgium	Final Mesolithic, early Neolithic	inductive	-	-	+	yes	EMI data, sediment-drilling data, CPT data, present DTM	c. 0.25 km^2^	*Verhegge et al*. *2017*
Vilnius, Lithuania	-	inductive	-	-	-	no	archival material, historical sources, cartographic and visual material, geological borehole data, geophysical data	2.6 km^2^	*Baubinienne et al*. *2015*
Sandy Flanders, Belgium	Medieval	inductive	+	+	++	yes	EMI data, archaeological excavations	0.2 km^2^	*De Smedt et al*. *2013*
Altmühl, Germany	Mid-Holocene	inductive	-	--	+	no	sediment drilling data	c. 0.2 km^2^	*Kirchner et al*. *2017*
Lausitz, Germany	Mesolithic	inductive	--	--	+	no	archaeological excavation, GPR data, Drone-DTM	c. 0.1 km^2^	*Schneider et al*. *2017*
Remote Oceania	1,500–1,000 BC	combined	+	-	+	(yes)	present DTM, sea level data (time series), archaeological data	few km^2^	*Carson 2014*
Pederneira lowland, Portugal	Pre-Holocene	combined	o	o	-	no	present DTM, geological borehole data, TEM data	c. 16 km^2^	*Lopez et al*. *2013*
Scheldt Polders, Belgium	Final Mesolithic, early Neolithic	combined	o	-	o	no	archaeological drilling data, EMI data, Seismic shear, Electrical resistivity imaging, CPTs	c. 0.5 km^2^	*Verhegge et al*. *2016*
Sandy Flanders, Belgium	10,000 BC	combined	--	--	+(+)	yes	Filtered DEM from Werbrouck et al. 2011, Holocene sediment data from literature and own field studies, c. 4000 drillings from geological database	584 km^2^	*Vermeer et al*. *2014*
Sandy Flanders, Belgium	-	combined	--	--	+	no	for temporal DEM -> present DTM	584 km^2^	*Zwertvaegher et al*. *2010*

Inductive approaches (Table 4) usually have an advantage in that the specific data points used for the interpolation are dated and have a stratigraphic context [30,48]. The spatial distribution of data points is critical. Geophysical methods can generate many spatially well-distributed data points [11,49,9]. The resolution and precision of the data depend on the sedimentary contrasts of the palaeo-surface and the overlaying sediments [50]. The amount of required fieldwork for obtaining and post-processing the data is time-consuming. In addition, the research area must be accessible or already explored, and archaeological, geological, or geoarchaeological data must be available.

Deductive approaches (Table 4) provide an advantage of handling the study area without laborious fieldwork and allow us to work with spatially well-distributed input data (DTMs, aerial images, etc.) that are relatively easy to obtain and well-achievable for large areas [16,10,17]. Usually, a present landscape is deconstructed via eliminating different structures with different techniques until the necessary state has been reached [10]. However, for a deductive approach, the temporal uncertainty of the modelled terrain and the validation of the result are critical. Van Loon et al. [51] used a deductive method and gave their model a chronological frame. This exception is due to their approach using peat subsistence ratios to model the altitude in five time slices. In the present study, we eliminated modern and specific historic features during our modelling approach. Therefore, we are able to provide at least a pre-modern stage as a chronological frame.

A combination of deductive and inductive approaches (Table 4) might improve the performance of the model. Usually, there is an approximation of the palaeo-surface with an deductive approach, which is subsequently supported by a dense dataset of archaeological or geoscientific ground-truth data [17,52,13]. Zwertvaegher et al. [17] developed an integrative process model to challenge the complexity of landscape evolution by combining a deductive approach to obtain a purged base layer and an inductive approach for the predictive modelling of archaeological finds within the landscape. The amount of data, their quality, and the effort are enormous and require a holistic understanding of the landscape. On the other hand, such integrative approaches provide a more accurate result than a deductive or inductive approach alone.

Our approach is deductive and characterized by easily obtained data (LiDAR-DTM, land use shapefiles, historical cadastre sheets, areal images), and therefore, it is well applicable at different scales. Unlike many other studies with a deductive approach [14,15,51], we are able to give an accuracy estimation of our model. We use a ground-truth validation approach based on discrete palaeo-surface data from drillings and excavations to provide values for model accuracy. The qualitative 2D validation documents the specific offset of the model at two specific points of our study area. Our semi-quantitative validation approach compares point subsets from Zielhofer et al. [28], Werther et al. [39], and Kirchner et al. [30] with the modelled terrain height. In addition, we measured the error between the different models, today’s DTM, and the modelled DTM and developed an improvement value. The validation results of our study show a spatial unequal distribution of the model error. In the Altmühl floodplain, which has a low anthropogenic impact (mainly planar accumulation of overbank fines), the improvement compared to the present DTM is negligible (see Table 3). On the other hand, in the proximal area of the *Fossa Carolina* construction site, the modelling of the pre-modern DTM significantly improves the accuracy.

### Model performance

The performance of the model is crucial for the reliability of the results and their interpretation. For the qualitative validation, drilling and model data were connected in a 2D plot, and model inaccuracies were estimated (see Figs 7 and 8). The offset of the modelled altitude and the detected palaeosols is low to moderate. However, if we consider the absence of a buried A-horizon and the thickness of the buried B-horizons [53], it is clear that these soils were truncated before the Carolingian construction phase [54]. Therefore, the “natural” offset should be lower since the original surface was higher. Furthermore, the height accuracy of the LiDAR-DTM with ±0.2 m is taken into account, and the semi-quantitative validation results are discussed.

No other deductive modelling approach has been validated with palaeo-pedological and stratigraphic field data (see Table 4). Only Werbrouck et al. [16] validated their model at all, but only based on a historic map from 1909 to correlate drawn and modelled ditches qualitatively. However, the detection of palaeosols is not flawless. Among others, one part of the described offsets may result from sediment loss and sediment consolidation during canal construction. Additionally, the region between Weissenburg and Treuchtlingen was used intensively since at least the Iron Age, resulting in thick pre-medieval colluvial and fluvial deposits around the *Fossa Carolina* [55].

Our approach only allows the removal of linear or punctual features of the model, so the main problem is erosion and accumulation with a wider spatial impact. This large-scale impact on the terrain is not clearly detectable with our modelling approach. On the other hand, our semi-quantitative validation does only show minor offsets. Furthermore, general relief characteristics remain undisturbed and suitable for hydrogeographic modelling approaches.

### Evidence of excellent Carolingian knowledge in engineering

The LCPA reveals the most cost-efficient course of *Fossa Carolina* and allows for a comparison of the modelled and the real Carolingian canal course. The overall shapes of both courses are nearly identical. The sharp bend framed by the west-east section and the northern section is thus mainly induced by the topography and not by geological conditions, as postulated by Koch [56]. The topography and the predicted earth volume must therefore have been the crucial factors in the process of decision making concerning the course of *Fossa Carolina*. Other factors may also have played a role but were not necessarily key, such as the possibility of connecting the Swabian Rezat with the canal in order to supply the summit section with water (as suggested by Zielhofer et al. [28]).

As illustrated in Fig 11, there is a small offset between the modelled course and the real Carolingian course in several sections. We assume that these shifts are related to the hydro-engineering concept and practical reasons of organisation of the construction site. The course of the canal within the depression is less laborious (with minimal excavation material), but it has to start under wet conditions from the very beginning. Accessibility and the transport of men, building material, and excavated material would have been much more difficult there. Furthermore, keeping the construction site drained and as “water-free” as possible was most likely a major task. In the depression line, Carolingian hydro-engineers would have been confronted with interflow and inflowing groundwater. Furthermore, the sediments in the wet depression are organic-rich loams and peats [29]. The stability of embankments of those sediments for canal construction is much more challenging.

Without using modern data and survey techniques, Carolingian engineers traced out the canal along the most effective route with an impressive level of precision. The deviations from the ideal line further underline that the people in charge had a deep understanding of the local topography and hydrology, as well as technical means to apply all that knowledge in a perfectly surveyed canal course. The course is thus a carefully chosen compromise between the minimum earthmoving and the maximum geotechnical stability and site accessibility.

## Conclusion

We have provided a general approach for the revision of high-resolution DTMs. The concept of the pre-modern DTMs is a well-reproducible prospecting method for geoarchaeological, historic-geographical, geomorphological, and palaeohydrological issues. The high spatial resolution offers the possibility of making even small-scale changes of the terrain visible. Since most of the input data is widely available, the transferability to other study sites is very high. The subtraction of the pre-modern model from the modern DTM generates a highly significant raster layer that visualises the local human impact on the relief. This dataset has high potential as a prospection and visualization tool for geoarchaeological issues.

For the first time, we presented a deductive modelling approach with a two-way validation using palaeo-pedological data to estimate the error of our model. Based on the reliable pre-modern DTM, we modelled the most favourable course of *Fossa Carolina*. The general modelled course is nearly identical to the Carolingian course. Slight deviations of the predicted course document a carefully chosen compromise between the minimum earthmoving and the maximum geotechnical stability and site accessibility. This suggests that the Carolingian engineers had an impressive understanding of the landscape, hydro-engineering, large-scale construction site organisation, and surveying.

## Supporting information

S1 Fig LiDAR-DTM of the study area. Illustrated with hillshade.
(PNG)

S2 Fig Comprehensive buffer layer of the study area. Underlain by LiDAR-DTM hillshade.
(PNG)

S3 Fig Local relief model of the study area. (PNG)

S4 Fig Pre-modern DTM of the study area. Illustrated with hillshade.
(PNG)

S5 Fig Topographic wetness index of the study area and the modelled and present course of the canal. (PNG)
