# Supplementary figures and images for: Shaping pre-modern digital terrain models: The former topography at Charlemagne’s canal construction site
Source: PLoS One. 2018 Jul 5;13(7):e0200167. doi: 10.1371/journal.pone.0200167 (PMC6033447; doi:10.1371/journal.pone.0200167)

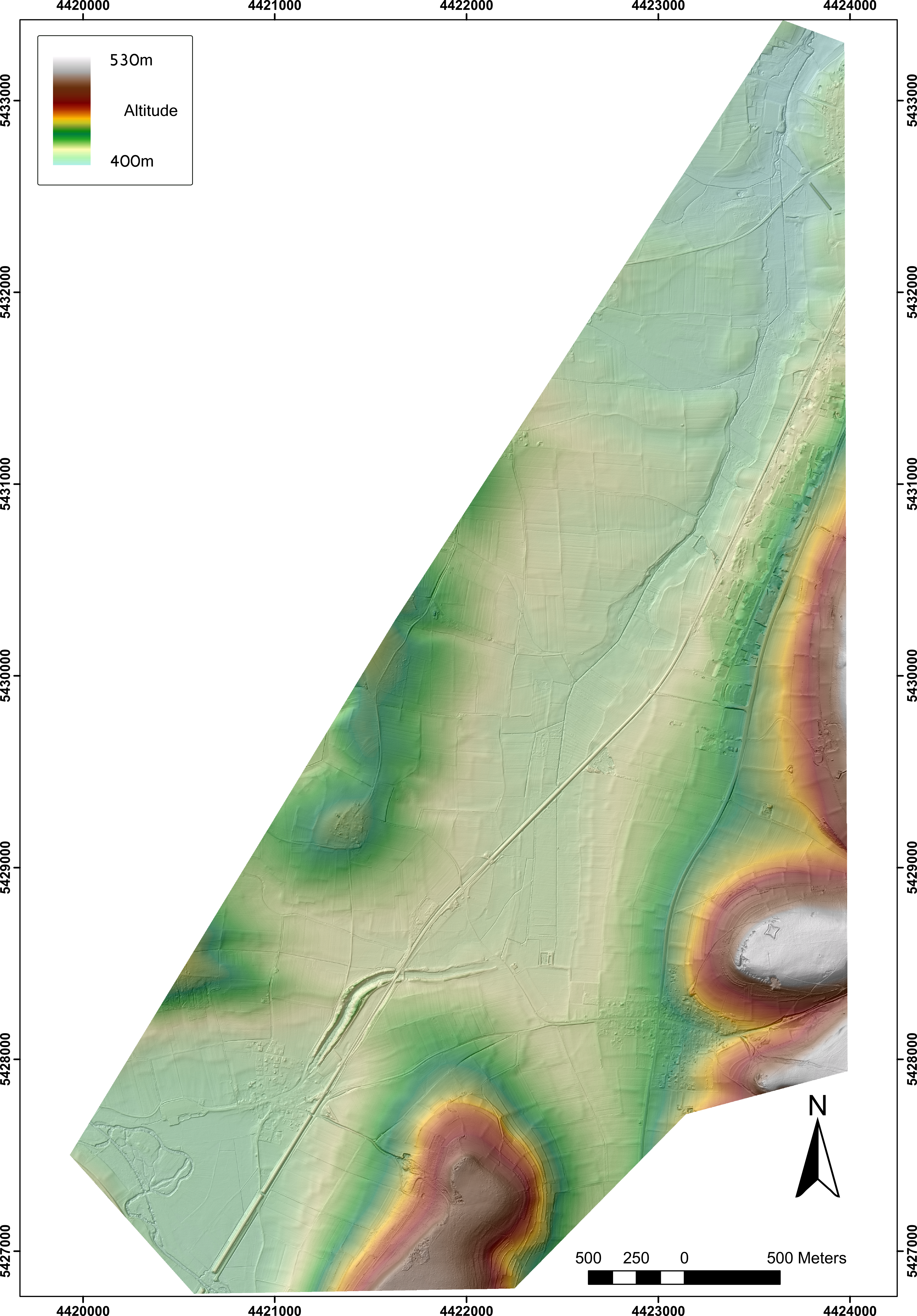

Supplement: S1 Fig — Illustrated with hillshade. (PNG) [file pone.0200167.s001.png]

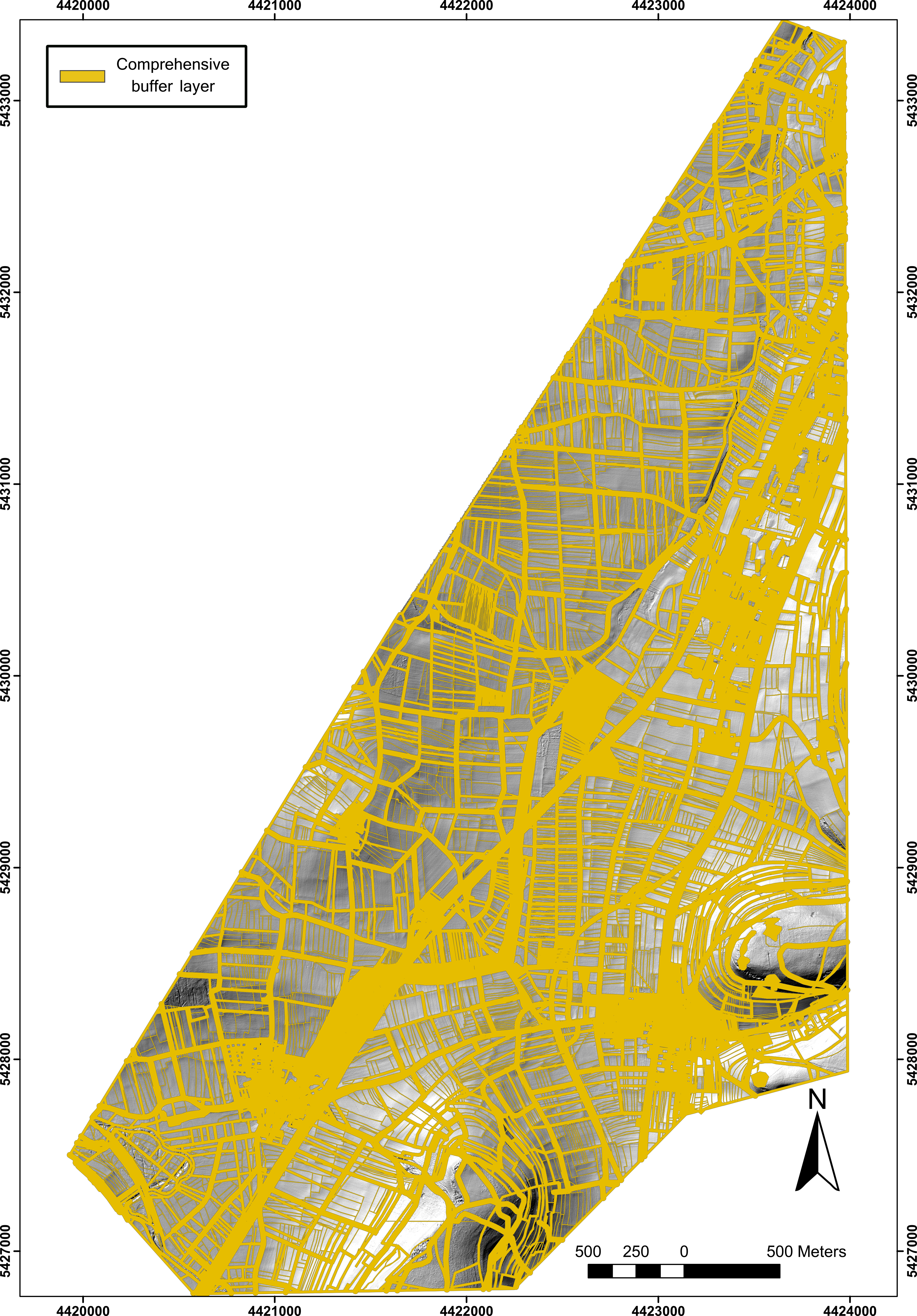

Supplement: S2 Fig — Underlain by LiDAR-DTM hillshade. (PNG) [file pone.0200167.s002.png]

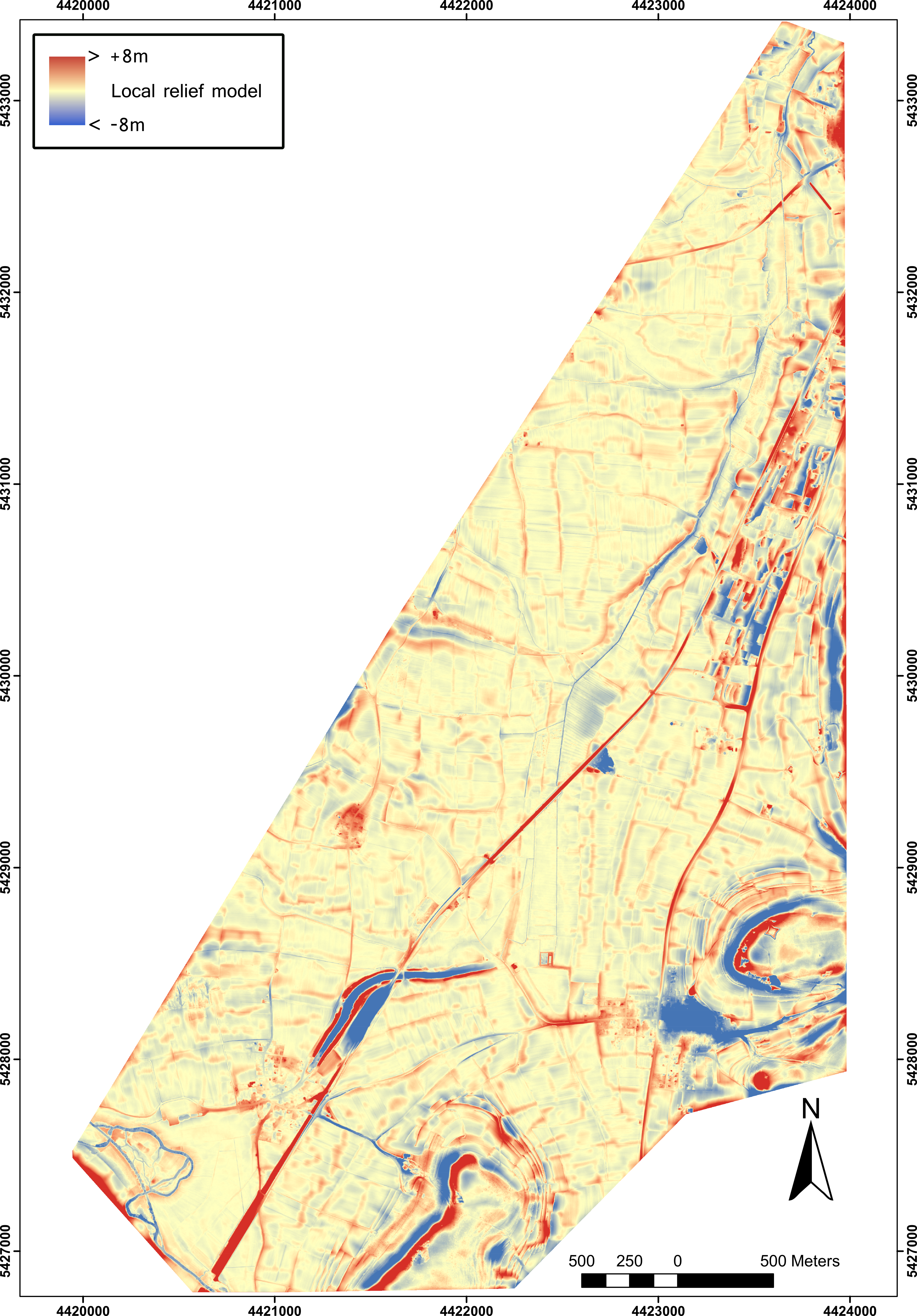

Supplement: S3 Fig — (PNG) [file pone.0200167.s003.png]

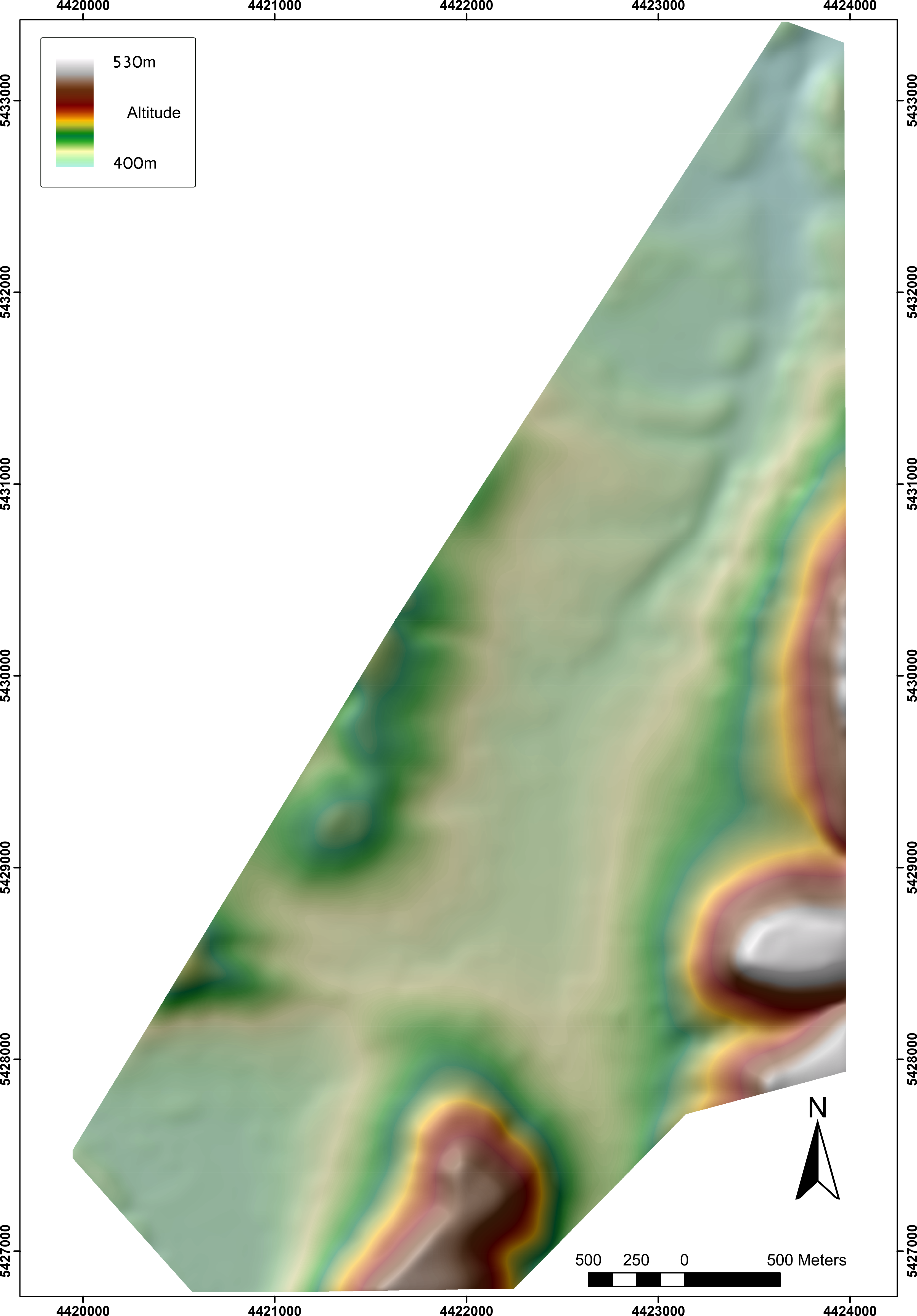

Supplement: S4 Fig — Illustrated with hillshade. (PNG) [file pone.0200167.s004.png]

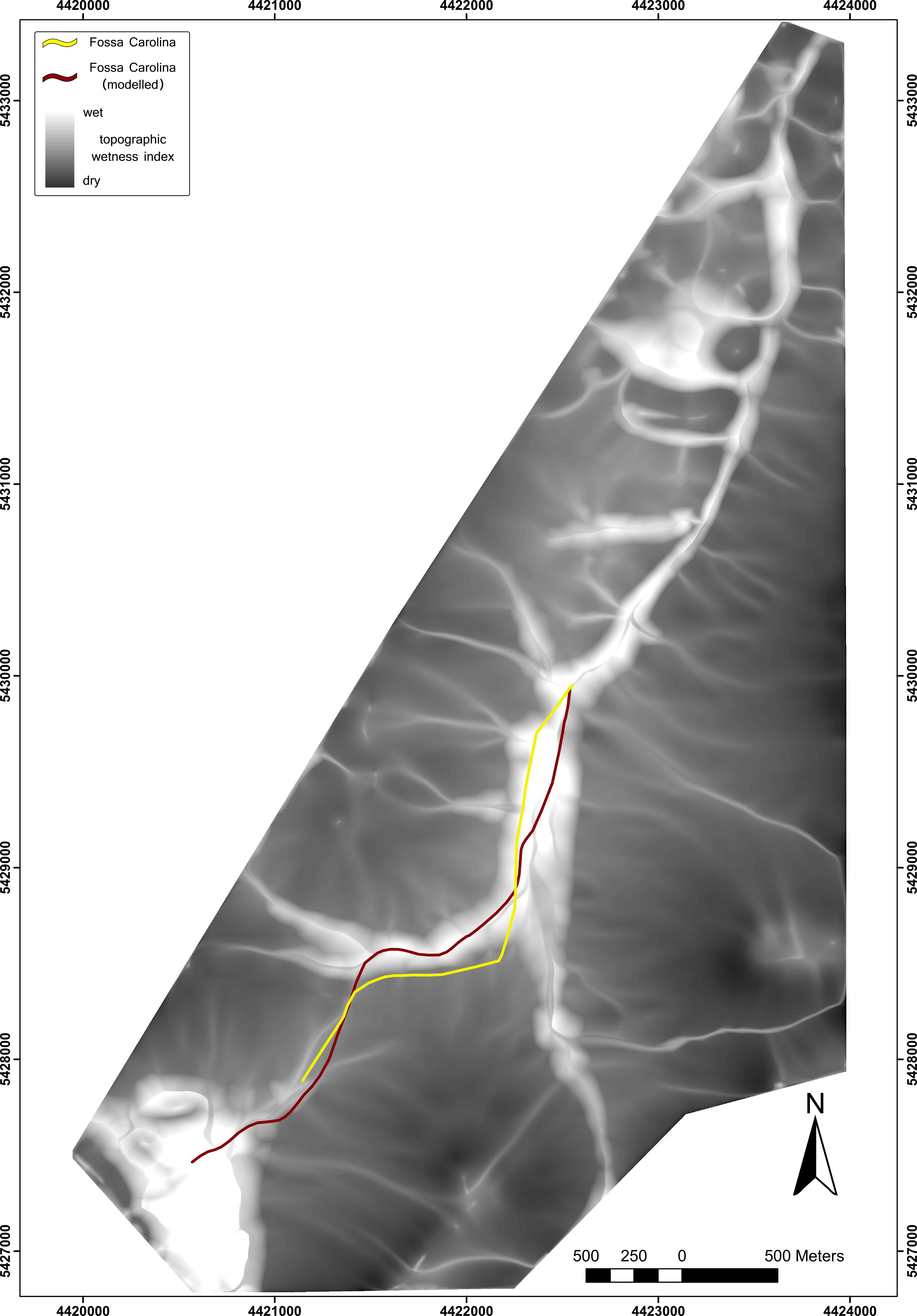

Supplement: S5 Fig — (PNG) [file pone.0200167.s005.png]
